# A Note on the Honey Bee Parasitic Phorid Fly (*Apocephalus borealis* Brues) in an Urban Ecosystem

**DOI:** 10.3390/insects16080765

**Published:** 2025-07-25

**Authors:** Lioh Jaboeuf, Miguel Cabrera, Jenny Hoffmann, Emma Gallagher, Laura Byrne, John F. Mejía, Mitzy F. Porras

**Affiliations:** 1 Department of Biology, San Francisco State University, 1600 Holloway Ave, San Francisco, CA 94132, USA; 2 School Specialized in Biotechnology Engineering, Institute SupBiotech, 94800 Villejuif, France; 3 Division of Atmospheric Sciences, Desert Research Institute, Reno, NV 89512, USA

**Keywords:** species interactions, host, parasitoid, seasons

## Abstract

The honey bee is a crucial pollinator in urban ecosystems but faces multiple challenges, including habitat degradation, pollution, and parasitism by species such as the phorid fly, *Apocephalus borealis* Brues (Diptera: Phoridae). This study investigated honey bee abundance and the percentage of *A. borealis* parasitism in an urban environment in San Francisco, California. We monitored six sites weekly for six months using two sampling methods. Individual bees were weighed and observed for parasitoid emergence under controlled laboratory conditions. Our results indicate fluctuations in honey bee parasitism by *A. borealis* from September 2024 to May 2025, with four distinct peaks occurring in mid-September, February, late March, and early May. The highest parasitism rates exceeded 50% in early May, coinciding with increased temperatures and drops in relative humidity. These results suggest a potential link between abiotic conditions and parasitoid activity, highlighting the importance of long-term monitoring to understand the seasonal dynamics of host–parasite interactions in urban environments.

## Simple Summary

Honey bees are crucial for pollinating plants, including those in urban areas. However, they face numerous threats, including habitat loss, pollution, and parasitic insects such as the phorid fly, which can infect bees and potentially harm them. In this study, we monitored the parasitism of worker honey bees by a phorid fly on the San Francisco State University Campus over two seasons. Bees were collected weekly and observed for parasite emergence. We found that the numbers of both parasitized and unparasitized bees varied over time, with notable peaks in late September and early May. Our findings indicate that while this parasitoid fly is present in the urban environment, it may not be a primary driver of honey bee mortality during the autumn and spring months.

## 1. Introduction

Honey bees are susceptible to multiple parasites, including bacteria, fungi, viruses, mites, and a phorid fly parasitoid, *Apocephalus borealis* Brues [1], which is distributed across Canada and the United States. In Canada, it has been recorded in Alberta, British Columbia, New Brunswick, Nova Scotia, Ontario, and Québec. In the US, specimen records are primarily concentrated along the East and West Coasts, including California, South Dakota, Oregon, Washington, Utah, New Mexico, Georgia, Tennessee, West Virginia, Virginia, Vermont, and Maine [2]. The species has also been reported in Belgium [2] and Egypt [3]. The parasitoid phorid fly attacks bumblebees, paper wasps, and honey bees by laying eggs on or within the host’s thorax. The phorid larva feeds on the muscles and hemolymph, ultimately killing the bee [4].

Phorid parasitism of pollinators became a concern in 2012, when it significantly increased the mortality of Western honey bees (*Apis mellifera*) in hives on the San Francisco State University campus. *Apis mellifera* was introduced to America in the 1600s [5] and has become a key pollinator for agricultural ecosystems [6]. This pollinator is also present in urban ecosystems. Studies have shown that urban areas may serve as refuges for honey bees, offering diverse floral resources and reduced pesticide exposure compared to agricultural landscapes [3,7,8]. However, urban environments also host feral colonies, and both managed and feral populations remain susceptible to stressors, including phorid fly parasitoids [4].

Parasitism by *A. borealis* represents an additional stressor contributing to Colony Collapse Disorder (CCD) in honey bees, which increases hive mortality and impairs the establishment of new hives [9,10]. Parasitoid infection impairs worker bee function, reducing foraging efficiency and colony maintenance, which compromises hive health. When coupled with other environmental stressors, pathogens, and pesticide exposure, *A. borealis* parasitism can accelerate colony decline, highlighting its potential role in the etiology of CCD [10].

North America is climatically suitable for *A. borealis* infestations, and with climate change, its range may expand, resulting in increased economic and environmental harm [9]. Coupled with expected urban expansion in the coming decades, the potential increase in the abundance and dispersal of the phorid fly parasitoid [5] should be monitored to better understand its ecological interactions and inform conservation strategies that support pollinator resilience in cities [6].

Here, we (1) examined temporal changes in honey bee abundance on the SFSU campus, (2) determined the prevalence of *A. borealis* parasitism in honey bees over 6 months in 2024 and 2025, and (3) evaluated the effects of parasitism on honey bee body mass. These objectives allowed us to assess how urban environmental conditions may influence both pollinator populations and their interactions with natural enemies across seasons. By integrating parasitism and weather observations, this study offers insights into the current prevalence of phorid fly parasitism and its potential implications for honey bee health and urban pollination services.

## 2. Materials and Methods

### 2.1. Study Site and Sampling Protocol

This study was conducted across the campus of the San Francisco State University, California, from September to October 2024, and from February to May 2025. Sampling transects spanned two different types of gardens (irrigated and non-irrigated) known to attract pollinators and included herbaceous and woody plants as well as plantings near a building where parasitized honey bees were previously collected [7]. We monitored six different sites (37°43′28.5″ N 122°28′36.5″ W; 36°43′27″ N 122°28′46″ W; 37°43′25″ N 122°28′46″ W; 37°43′27″ N 122°29′04″ W; and 37°43′33″ N 122°28′56″ W) and the Hensill Hall building. We sampled a total area of 4138 m^2^ across five belt transects (transect 1 = 382 m^2^, transect 2 = 724 m^2^, transect 3 = 1171 m^2^, site 4 = 1329 m^2^, and transect 5 = 577 m^2^). Transect sizes varied due to differences in the shape and layout of the gardens on the SFSU campus. We conducted sampling using the actual dimensions of each transect to accurately reflect the environmental conditions of the campus gardens in our study. Honey bee workers primarily from feral hives were collected every week from September to November 2024 and from February to early May 2025 between 9:00 and 11:00 a.m. We collected honey bees using insect nets (Bioquip, Monterey, CA, USA) and by hand. Bees were transferred into labeled vials indicating the date, time, location, and collector initials. Each individual bee was assigned a unique identifier code. Immediately after collection, bees were taken to the lab and anesthetized by placing them in a freezer at −15 ± 1 °C for 2–3 min. Then, we weighed each honey bee using an analytical scale (Ohaus, Pine Brook, NJ, USA and Mettler Toledo AX204, Zurich, Switzerland). Each honey bee was then placed in an individual plastic container with moistened soil at the base. Containers were covered with fine mesh secured by elastic bands to ensure adequate air circulation and incubated at 23 °C under controlled laboratory conditions for two weeks to allow for parasitoid pupation and the emergence of adult flies.

### 2.2. Statistical Analysis

All data were tested for statistical test assumptions using a qqplot, Levene’s homogeneity test and the Shapiro–Wilk normality test at alpha = 0.05 significance level. The effects of temperature, humidity, and their interaction on honey bee abundance were evaluated using a generalized linear model (GLM) with a Poisson distribution. Weather data were collected from the GW0189 San Francisco weather station located at latitude 37°43′7″ N, longitude 122°27′13″ W, elevation 129 m, accessed via the Synoptic environmental data platform, which provides data at hourly resolution. Values were extracted at 10 a.m. on each day of the sampling months to match the timing of bee collections. The body weight data sets of parasitized and unparasitized honey bees were not normally distributed, and data transformation did not normalize the residuals. Therefore, we used a non-parametric Wilcoxon test to compare the body weights of parasitized and unparasitized bees and assess the effects of parasitism. All statistical analyses were conducted using R (v. 3.4.3, CRAN project) [11].

## 3. Results

We sampled a total of 262 honey bees across 37 sampling dates from September 2024 to May 2025 on the San Francisco State University campus. Bee captures increased through late September and early October, reaching a local maximum of 17 individuals on 24 September. The highest numbers were recorded in late spring, with peaks of 19 and 15 bees on 1 May and 5 May, respectively (Table 1). Increases in honey bee abundance were significantly affected by rising environmental temperatures (*χ*^2^ = 27.17, DF = 3, *p* ≤ 0.0001; Table 2 and Table 3).

**Figure 1:**
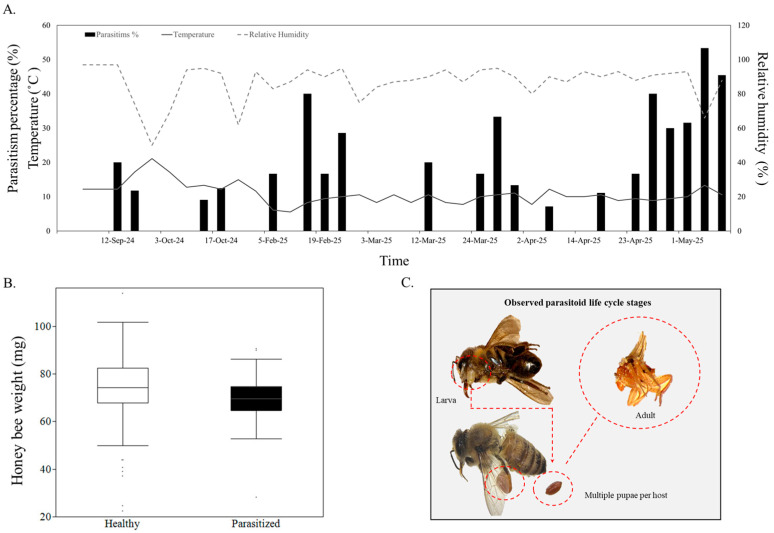
Parasitism of *Apis mellifera* by *Apocephalus borealis*. (**A**) Fluctuation in parasitism percentage in an urban environment over time. Time series in *A. borealis* parasitism, temperature (solid line, left *y*-axis), and relative humidity (dashed line, right *y*-axis) over autumn and spring. (**B**) Body weight (mg) of unparasitized and parasitized honey bees. Boxplots display median line, interquartile range (IQR) boxes, and 1.5 × IQR whiskers. (**C**) Parasitized honey bees and *A. borealis* larvae and pupae. The inset highlights the emergence of *A. borealis* larvae from a parasitized honey bee, the presence of multiple pupae within a single host, and the adult stage of the parasitoid.

The percentage of parasitism varied across the seasons, with at least three distinct peaks. An initial moderate peak was observed in mid-September 2024 and February 2025, oscillating between 20 and 40%, followed by a second increase in late March, with approximately 35%. The most pronounced and sustained rise occurred from mid-April through early May, a peak of over 50% by 5 May 2025 (Figure 1A). This final rise in parasitism coincided with an increase in mean temperature and a sharp drop in relative humidity. Unparasitized bees were heavier (74.20 ± 0.84 mg) than parasitized bees (69.82 ± 1.49 mg). There was a significant difference in the weight of parasitized and unparasitized bees at the time of capture (*W* = 4603, *p* = 0.0045; Figure 1B).

## 4. Discussion

Our study showed the expected seasonal fluctuations in honey bee activity, with low captures in early September and mid-March and peaks in late September and early May. These trends are consistent with temporal patterns in floral resource availability and temperature. The highest abundance recorded in May suggests increased foraging activity during spring bloom, while the absence of bees on certain dates may reflect unfavorable weather, such as rainstorms.

The percentage of parasitism of honey bee workers by *A. borealis* varied over the seasons, displaying three distinct seasonal peaks. These seasonal dynamics suggest that abiotic factors, such as temperature and relative humidity, may influence host–parasitoid interactions, either by affecting parasitoid activity or host susceptibility. Our findings are consistent with previous studies on bee parasitoids, which reported parasitism rates of approximately 20% in worker honey bees [12]. Seasonal variation in parasitism has been documented in other bee–parasitoid systems. For example, conopid flies that parasitize bumble bees exhibit pronounced seasonal patterns in infection rates, which are closely aligned with host phenology and environmental conditions [13]. These patterns across systems suggest that seasonal cycles are a major factor influencing the dynamics of parasitism in pollinator communities.

We also found that parasitism by *A. borealis* significantly reduced host body mass, indicating a substantial physiological cost. This reduction likely reflects tissue degradation and muscle atrophy associated with larval development, as has been observed in bumble bees parasitized by conopid flies [13,14]. However, additional research is needed to evaluate whether weight differences emerge in later stages of infection or affect energy expenditure. Ample sampling, considering variable regular time increments and all-weather parameters, is needed to improve robustness and representativity in the analysis [14,15].

Given the rapid expansion of urban landscapes, it is critical to investigate how multiple stressors interact to impact pollinator health and survival. Future research should examine how factors such as drought and extreme temperatures, including heat island effects, affect host–parasite dynamics in urban areas. This is particularly important as cities continue to transform ecological processes key for pollination [7]. Research efforts are critical to assess the long-term impact of *A. borealis* parasitism on honey bee colony health. For example, studies integrating pathogen screening, weather monitoring, and behavioral analysis could provide insights into the broader ecological implications of this emerging threat to managed and wild pollinators.

## Figures and Tables

**Table 1:** The number of honey bees collected per sampling date at the San Francisco State University campus from September 2024 to May 2025.

Date (m/d/y)	Number of Honey Bees	Temperature (°C)	Relative Humidity (%)
9/1/2024	6	12.22	97
9/5/2024	6	12.22	97
9/12/2024	5	12.22	97
9/24/2024	17	17.22	74
10/1/2024	16	21.11	50
10/3/2024	2	17.22	69
10/10/2024	4	12.78	94
10/15/2024	11	13.33	95
10/17/2024	8	12.22	92
10/24/2024	3	15	62
10/31/2024	1	11.67	93
2/5/2025	6	6.11	83
2/12/2025	4	5.56	87
2/17/2025	5	8.33	94
2/19/2025	6	9.44	90
2/24/2025	7	10	95
2/26/2025	6	10.56	75
3/3/2025	1	8.33	84
3/5/2025	6	10.56	87
3/10/2025	1	8.33	88
3/12/2025	10	10.56	90
3/17/2025	0	8.33	94
3/19/2025	0	7.78	87
3/24/2025	6	10	94
3/26/2025	6	10.56	95
3/31/2025	15	11.11	90
4/2/2025	0	7.78	80
4/7/2025	14	12.22	90
4/9/2025	2	10	87
4/14/2025	6	10	93
4/16/2025	9	10.56	90
4/21/2025	6	8.89	93
4/23/2025	6	9.44	88
4/28/2025	5	8.89	91
4/30/2025	10	9.44	92
5/1/2025	19	10	93
5/5/2025	15	13.33	66
5/7/2025	11	10.56	88

**Table 2:** Effects of temperature and relative humidity on *Apis mellifera* abundance on San Francisco State University campus (Acc: 254.01).

Source	DF	L-R ChiSquare	Prob > ChiSq
Temperature	1	16.030752	<0.0001
Relative Humidity	1	0.8445738	0.3581
Relative Humidity × Temperature	1	0.080062	0.7772

**Table 3:** Parameter estimates of analysis of deviance (type II) from the study testing the effects of temperature and relative humidity on *Apis mellifera* abundance on the San Francisco State University Campus.

Term	Estimate	Std Error	L-R ChiSquare	Prob > ChiSq	Lower CL	Upper CL
Intercept	−6.947936	5.7098932	1.3748912	0.2410	−17.48272	4.9573346
Temperature	0.7802166	0.1799716	16.030752	<0.0001	0.4121904	1.1166495
Relative Humidity	0.0603672	0.0641885	0.8445738	0.3581	−0.071724	0.180122
(Relative Humidity—86.9474) × (Temperature—10.8916)	−0.003857	0.0137284	0.080062	0.7772	−0.032224	0.0219167

## Data Availability

The original data presented in the study are openly available in the figshare data repository at https://doi.org/10.6084/m9.figshare.29441909.v1.
